# Let-7b Is Involved in the Inflammation and Immune Responses Associated with *Helicobacter pylori* Infection by Targeting Toll-Like Receptor 4

**DOI:** 10.1371/journal.pone.0056709

**Published:** 2013-02-20

**Authors:** Gui-gen Teng, Wei-hong Wang, Yun Dai, Shu-jun Wang, Yun-xiang Chu, Jiang Li

**Affiliations:** Department of Gastroenterology, Peking University First Hospital, Beijing, China; Vanderbilt University Medical Center, United States of America

## Abstract

**Objectives:**

Toll-like receptors (TLRs) are important initiators in native immune responses to microbial infections. TLR4 is up-regulated in response to *H.pylori* infection in gastric epithelial cells. However, the regulatory mechanisms for the expression of TLR4 in *H.pylori* infection have not been clearly defined. The aims of this study are to present the evidence that microRNA let-7b directly regulates TLR4 expression in human gastric epithelial cells, and subsequently influences the activation of NF-κB and the expression of the downstream genes in *H.pylori* infection.

**Methods:**

The expression of let-7b was determined in gastric mucosa specimens and in two gastric epithelial cell lines using quantitative RT-PCR. The expression of TLR4 was determined by immunohistochemistry staining and RT-PCR. The potential target of let-7b was identified by luciferase reporter assay and Western blot. Let-7b mimics and inhibitors were used to examine the effects of let-7b on NF-κB activity. The expression of the downstream genes of NF-κB was also determined in cells infected with *H.pylori* 26695.

**Results:**

Let-7b was significantly decreased in gastric mucosa specimens and in gastric epithelial cell lines (AGS, GES-1) infected with *H.pylori* 26695 (cagA+). Let-7b was complementary to the 3′-UTR of TLR4 mRNA and regulated TLR4 expression via post-transcriptional suppression in gastric epithelium. Infection of *H.pylori* induced the expression of TLR4 and activated NF-κB in AGS and GES-1 cells. Overexpression of let-7b by mimics downregulated TLR4, and subsequently attenuated NF-κB, MyD88, NF-κB1/p50, RelA/p65. The expression of IL-8, COX-2 and CyclinD1 was inhibited in *H.pylori* infected cells with let-7b overexpression. Both TAK-242 (TLR4 inhibitor) and SN50 (NF-κB inhibitor) significantly inhibited the *H.pylori* induced downregulation of let-7b.

**Conclusions:**

Let-7b targets at TLR4 mRNA, and regulates the activation of NF-κB and the expression of the downstream genes related to the inflammation and immune responses in *H.pylori* infection.

## Introduction

*Helicobacter pylori* is a Gram-negative microaerophilic bacterium infected with more than 50% human gastric mucosa and is believed to be the main causative factor of chronic gastritis, peptic ulcer disease and gastric adenocacinoma. Infection of *H.pylori* causes strong immune responses and chronic inflammation of gastric mucosa. The outcomes of *H.pylori* infection are closely related to the virulence of bacteria, the host immune responses and the environmental factors. As the first barrier against bacteria invasion, gastric epithelial cells initiate the natural immunity of gastric mucosa by expressing Toll-like receptors (TLRs) after *H.pylori* colonization [1]–[3]. Previous studies indicate that the expression of TLR4 in gastric epithelial cells activates myeloid differentiation protein 88 (MyD88) leading to NF-κB activity and the production of cytokines/chemokines, which causes chemotaxis of monocytes/macrophages and polymorphonuclear leukocyte infiltration [4], [5]. Therefore, gastric epithelial cells play an important role in the immune responses and chronic inflammation of gastric mucosa. TLR4 expression is involved in controlling the immune responses and inflammation of gastric mucosa associated with *H.pylori* infection. Expression of TLR4 by epithelia needs to be tightly regulated to ensure that it does not cause an inappropriate immune response against endogenous ligand or symbiotic microorganisms [6]. However, the regulatory mechanisms for the expression of TLR4 in *H.pylori* infection have not been clearly defined.

MicroRNAs (miRNAs) are a class of about 18–25 nucleotides noncoding RNAs that control up to 60% protein-encoding gene expression at the post-transcriptional level by binding to 3′-UTR of target messenger RNAs (mRNAs) and initiating either their cleavage or translational suppression [7]–[9]. MiRNAs are involved in the regulation of various physiological and pathological processes, such as cell proliferation, differentiation and apoptosis [10], [11]. In recent years, studies have shown that miRNAs are indispensable molecules involving in the generation and differentiation of immune cells and mediating innate and acquired immune responses [12]. MiRNAs can implement a quantitative regulation to target genes. Studies indicate that they are fine-tuners of TLR signaling [13]. TLR2 protein has been shown to be regulated by miR-105 in human oral keratinocytes [14]. MiRNA-223 is found to be a strong candidate in regulating both TLR3 and TLR4 expression [15]. Let-7e decreases the expression of TLR4 on the surface of mouse peritoneal macrophages [16]. Downregulation of let-7i expression is revealed to upregulate TLR4 protein in human cholangiocyte after Cryptosporidium parvum (C.parvum) infection [17]. Some miRNAs change rapidly when cells infected with certain microorganism to regulate the expression of TLRs and control the intensity of immune responses at the initial stage; while others are regulated by molecules of the signal pathways of innate immune, and subsequently influence the expression of the downstream genes to fine regulate the immune responses [12].

Persistent infection with *H.pylori* probably induces chronic inflammation of gastric mucosa and even gastric adenocarcinoma via altering the expression of miRNAs in gastric epithelial cells. However, little is known regarding on the role of miRNAs in regulating the immune responses and inflammation in *H.pylori* infection. Studies indicate that the expression of miR-155 is increased in patients with *H.pylori* infection and in gastric epithelial cells *in vitro*. Overexpression of miR-155 negatively regulates the release of IL-8 and growth-related oncogene(GRO)–α [18]. This negative regulation of *H.pylori* induced inflammation is achieved by regulating MyD88 transcription [19]. Another study reveals that miR-146a targets IL-1 receptor-associated kinase 1 (IRAK1) and TNF receptor-associated factor 6 (TRAF6), and negatively modulates *H.pylori*-triggered expression of IL-8, growth-related oncogene(GRO)-α and macrophage inflammatory protein (MIP)-3α by diminishing NF-κB activity in gastric epithelial cells [20]. These findings indicate that miRNAs in gastric epithelial cells are involved in regulating the innate immune responses against *H.pylori* infection.

Let-7 is known as a tumor suppressor miRNA participating in cell differentiation, proliferation and apoptosis [21], [22]. Downregulation of let-7 has been found in several cancers, including gastric adenocacinoma [23], [24]. Recently, Matsushima et al. found that let-7 family (let-7a, let-7b, let-7d, let-7e, let-7f) were significant decreased in patients with *H.pylori* infection using a microarray method [25]. We screened the targets of let-7b by using Targetscan (version 6.0, www.targetscan.org) and found that TLR4 might be a putative target gene of let-7b. However, the role of let-7b in regulating TLR4 and the influence on the downstream gene expression in gastric epithelial cells in *H.pylori* infection has not been understood. Therefore, we investigated the expression of let-7b in response to *H.pylori* infection, and explored the possibility of let-7b in regulating the expression of TLR4. Our results suggest that let-7b regulates TLR4 expression in gastric epithelial cells and contributes to the epithelial immune responses against *H.pylori* infection.

## Results

### Let-7b is Significantly Decreased in Response to cagA Positive *H.pylori* Infection

**Figure 1:**
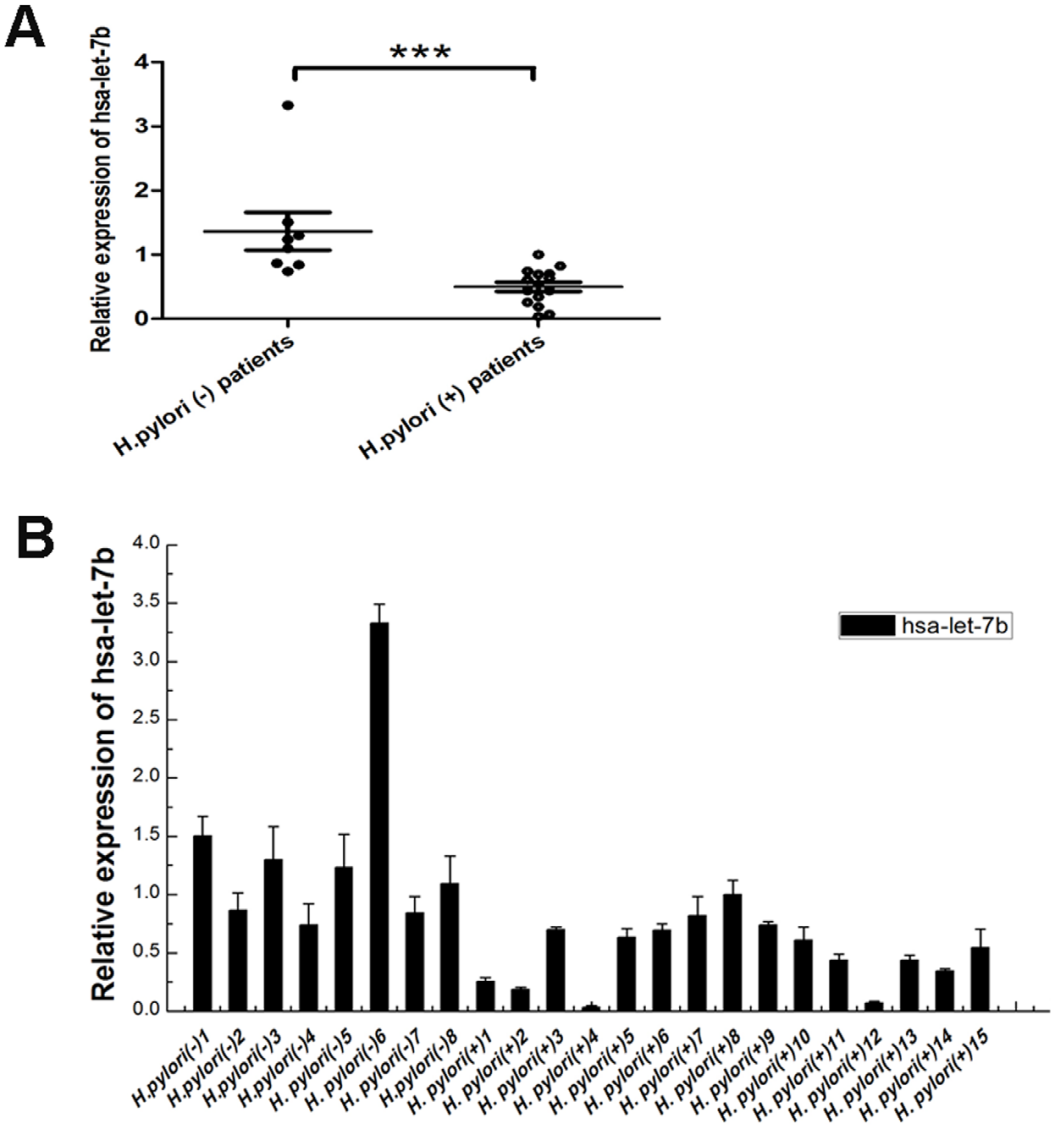
Let-7b is significantly decreased in gastric mucosal specimens infected with *H.pylori*. (A) Relative expression of let-7b in gastric mucosal tissues from *H.pylori*-positive patients (n = 15) and *H.pylori*-negative normal individuals (n = 8). (B) Relative expression of let-7b in individual gastric mucosal specimen. Relative expression levels of let-7b were determined by qRT-PCR. All data were normalized by U6 snRNA. Data were expressed as mean ± S.D. from three independent experiments. ***p<0.001.

**Figure 2:**
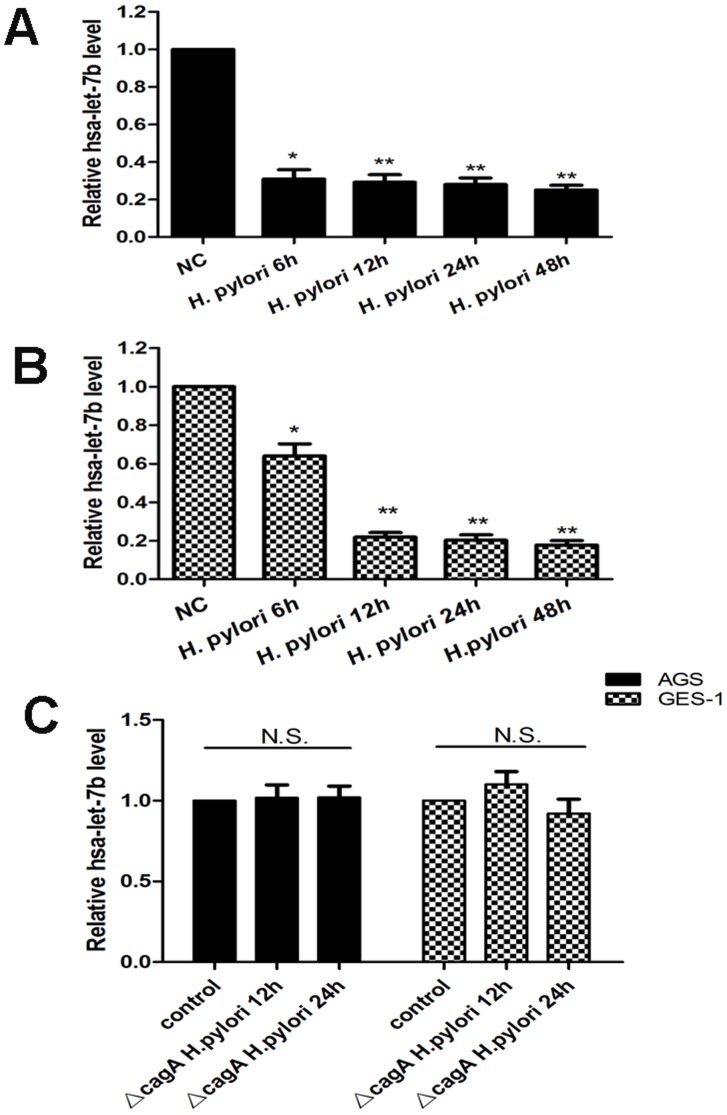
Let-7b is significantly decreased in response to cagA positive *H.pylori* infection in AGS and GES-1 cell lines. (A) Relative let-7b expression in AGS cells after *H.pylori* infection. (B) Relative let-7b expression in GES-1 cells after *H.pylori* infection. (C) Relative let-7b expression in AGS and GES-1 cells with ΔcagA *H.pylori* infection. Relative expression levels of let-7b were determined by qRT-PCR. All data were normalized by U6 snRNA. Data were expressed as mean ± S.D. from three independent experiments. *p<0.05, **p<0.01.

We measured the levels of let-7b expression in biopsy specimens from 15 patients with *H.pylori*-induced gastritis and 8 *H.pylori*-negative controls. All specimens from *H.pylori*-induced gastritis revealed significant infiltration of neutrophiles and lymphocytes, whereas *H.pylori*-negative controls had almost normal mucosa. Quantitative real-time PCR revealed that let-7b was significantly decreased in gastric mucosa with *H.pylori*-induced gastritis than in *H.pylori*-negative controls (Figure 1A, 1B). To confirm the validity of the results, we measured the expression of let-7b in a human gastric adenocarcinoma cell line (AGS) and a nonmalignant gastric epithelial cell line (GES-1) *in vitro*. We found that let-7b was also down-regulated in *H.pylori* 26695 infection (Figure 2A, 2B). In order to determine the role of cagA status of *H.pylori* for the downregulation of let-7b expression, a cagA mutant strain of *H.pylori* 26695 (ΔcagA 26695) was used. As shown in Figure 2C, ΔcagA *H.pylori* could not decrease the expression of let-7b in AGS and GES-1 cells. The findings demonstrate that the expression of let-7b is decreased in response to cagA positive *H.pylori* infection.

### Expression of TLR4 is Up-regulated in Response to *H.pylori* Infection in Gastric Mucosa Specimens and Cell Lines

**Figure 3:**
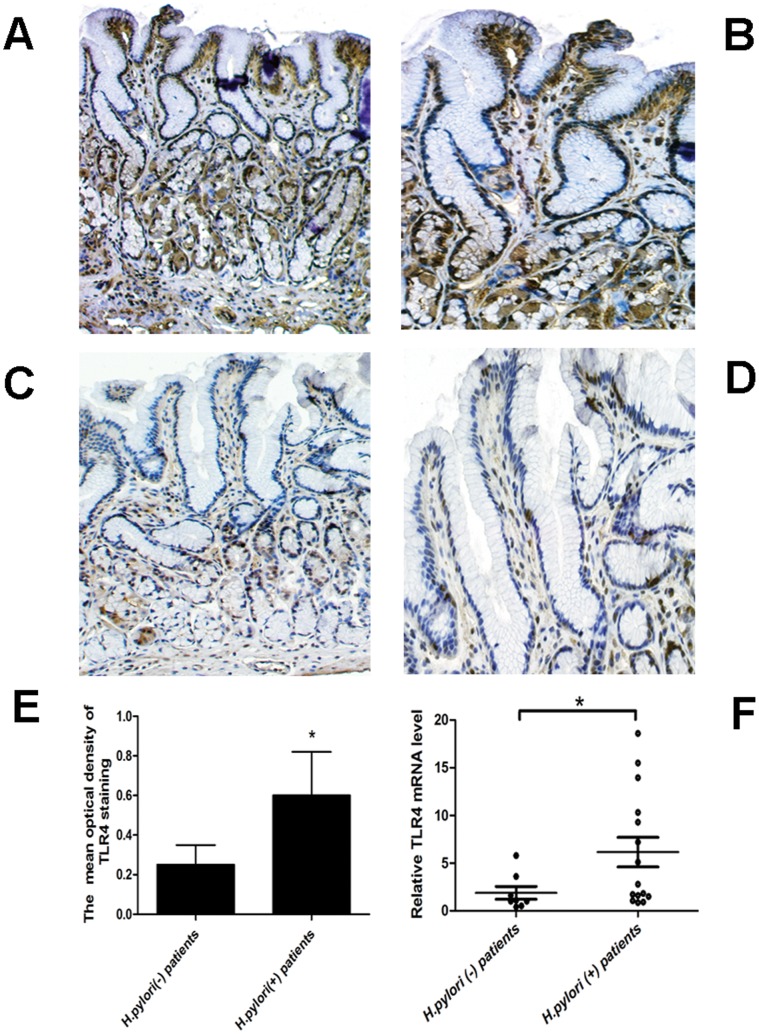
TLR4 expression is significantly increased in gastric mucosal specimens infected with *H.pylori*. (A–B) Gastric mucosa with *H.pylori* infection. TLR4 was expressed both in lamina propria cells and in the cytoplasm of gastric epithelial cells. (C–D) *H.pylori*-negative mucosa. TLR4 was expressed mainly in lamina propria cells. (E) The mean optical density of TLR4 staining in gastric mucosal specimens from *H.pylori*-positive patients (n = 15) and *H.pylori*-negative normal individuals (n = 8). (F) Relative expression of TLR4 mRNA in gastric mucosal specimens. Magnification: A, C, 100×; B, D, 400×. *p<0.05.

Immunohistochemical analysis for TLR4 was performed on paraffin sections of antral biopsy specimens obtained from 15 patients with *H.pylori*-induced gastritis and 8 *H.pylori*-negative controls. In gastric mucosa with *H.pylori* infection, TLR4 was expressed both in lamina propria cells and in the cytoplasm of gastric epithelial cells (Figure 3A, 3B). In *H.pylori*-negative mucosa, TLR4 was expressed mainly in lamina propria cells (Figure 3C, 3D). The mean optical density of TLR4 staining in patients with *H.pylori*-induced gastritis was significantly higher than that in normal controls (Figure 3E). The expression levels of TLR4 mRNA were also increased in gastric mucosa specimens with *H.pylori*-induced gastritis than in *H.pylori*-negative controls (Figure 3F).

**Figure 4:**
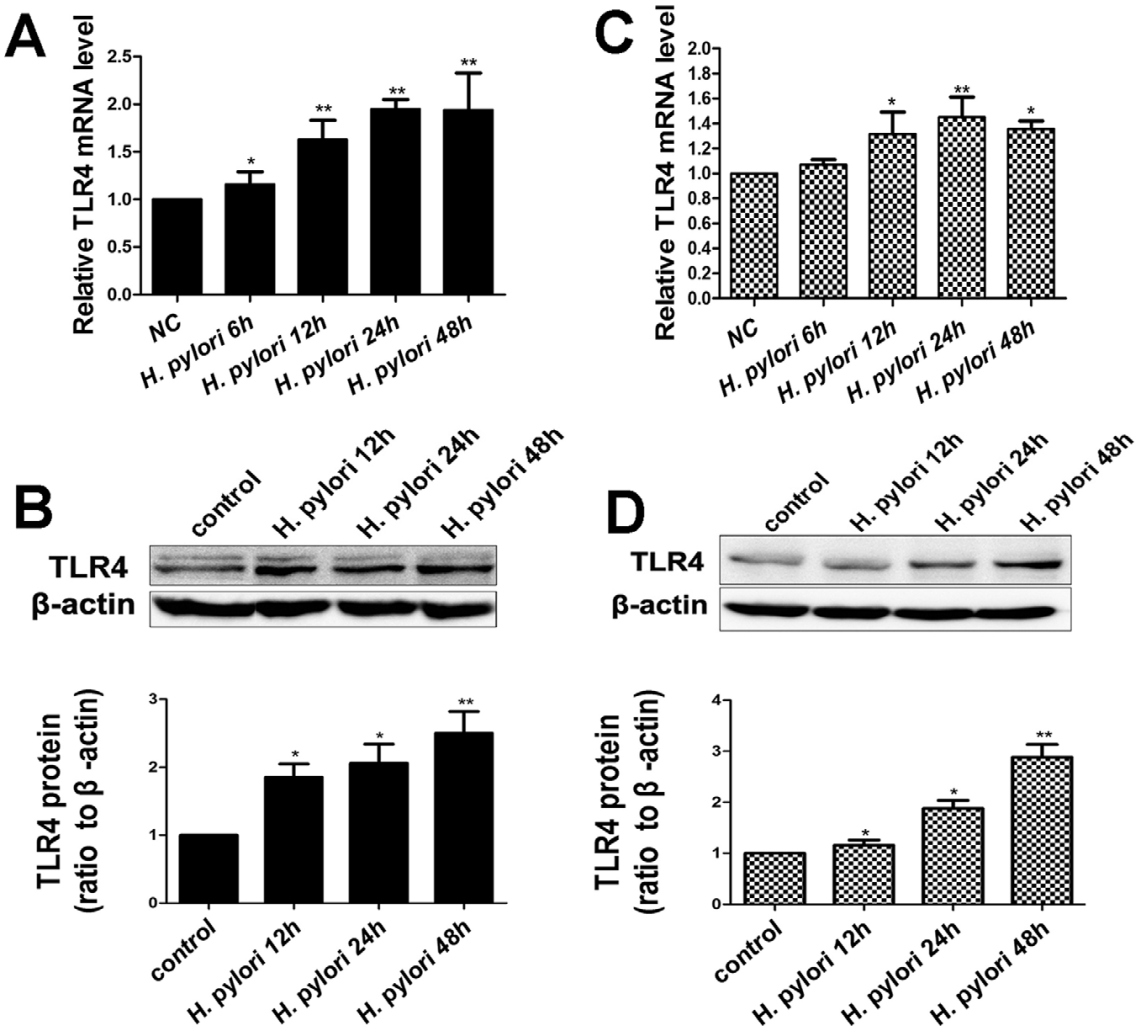
Expression of TLR4 is up-regulated in response to *H.pylori* infection. (A) *H.pylori* infection induced the transcription of TLR4 in AGS cells. (B) *H.pylori* infection induced TLR4 protein expression in AGS cells. (C) *H.pylori* infection induced the transcription of TLR4 in GES-1 cells. (D) *H.pylori* infection induced TLR4 protein expression in GES-1 cells. Total RNA was extracted from AGS and GES-1 cells following infection with *H.pylori* 26695 for 6 h, 12 h, 24 h, 48 h and analyzed by real-time PCR. All data were normalized by GAPDH. Western blot analyses were performed using total protein lysates of AGS and GES-1 cells. Data were expressed as mean ± S.D. from three independent experiments. *p<0.05, **p<0.01.

In *vitro*, TLR4 expression was measured in AGS and GES-1 cells after exposure to wild type *H.pylori* 26695 for 6 h, 12 h, 24 h and 48 h. As shown in Figure 4, AGS and GES-1 cells constitutively expressed TLR4. *H.pylori* infection induced the increased expression of TLR4 mRNA and protein at different time points.

### TLR4 is a Target of let-7b

**Figure 5:**
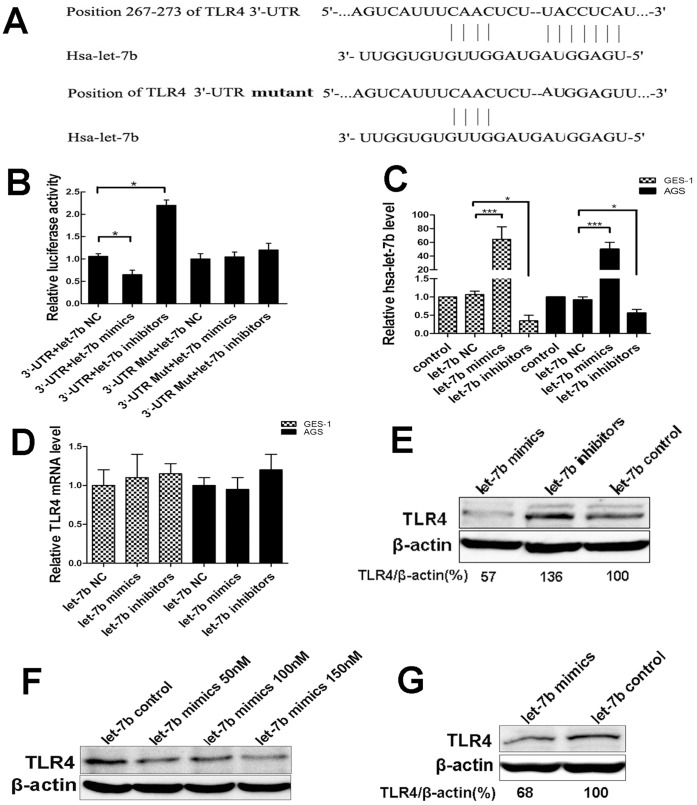
Let-7b targets at TLR4 and regulates TLR4 expression via post-transcriptional suppression. (A) The region of human TLR4 3′-UTR (wild type or mutant) predicted to be targeted by let-7b and mature sequence of let-7b. (B) Luciferase activity of the wild-type 3′-UTR or the mutant 3′-UTR of TLR4 with and without let-7b mimics treatment. HEK-293T cells were transiently cotransfected with luciferase report vectors (TLR4-3′-UTR and the mutant TLR4-3′-UTR), and either let-7b mimics or let-7b inhibitors. Relative luciferase activities were measured and normalized to control firefly luciferase level. (C–D) AGS and GES-1 cells were transfected with let-7b negative control, let-7b mimics, let-7b inhibitors at 100 nM for 24 h followed by quantitative RT-PCR for let-7b and TLR4 mRNA levels. (E–F) AGS cells were transfected with let-7b NC, let-7b mimics (50 nM, 100 nM and 150 nM), let-7b inhibitors for 48 h followed by Western blot for TLR4. A dose-dependent decrease of TLR4 protein was determined with let-7b mimics treatment. Treatment with let-7b inhibitors increased TLR4 protein. (G) TLR4 protein was decreased in GES-1 cells with let-7b mimics transfection compared to let-7b NC. Data were expressed as mean ± S.D. from three independent experiments. *p<0.05, **p<0.01, ***p<0.001.

We screened the targets of let-7b using Targetscan (version 6.0, www.targetscan.org) and found that TLR4 might be a potential target gene of let-7b. In Figure 5A, the potential binding sites was shown. Furthermore, we generated two luciferase report vectors containing the putative let-7b binding sites within TLR4-3′-UTR and the mutant TLR4-3′-UTR. As shown in Figure 5B, when cells transfected with the TLR4-3′-UTR vector, the relative luciferase activity was significantly decreased (p<0.05) in cells cotransfected with let-7b mimics compared to let-7b negative control. When cotransfected with let-7b inhibitors, the relative luciferase activity was significantly increased (p<0.05) compared to let-7b negative control. However, there was no difference in luciferase activity between cells cotransfected with let-7b mimics, let-7b inhibitors and the negative control when cells transfected with the mutant TLR4-3′-UTR vector. These results suggest that let-7b targets the predicted sites of TLR4 mRNA.

### Let-7b Regulates TLR4 Expression via Post-transcriptional Suppression

MiRNAs decrease the expression of target genes through mRNA cleavage or post-transcriptional suppression. In order to clarify the mechanisms resulting in the suppression of TLR4 expression by let-7b, we transfected AGS and GES-1 cells with let-7b mimics, let-7b inhibitors or let-7b NC (Figure 5C) and measured the mRNA levels of TLR4 by quantitative RT-PCR. The relative level of let-7b overexpression from let-7b mimics transfection is 64.5 times higher compared to endogenous level of let-7b in AGS cells, and is 50.1 times higher in GES-1 cells (Figure 5C). No significant difference was found in TLR4 expression at mRNA level (Figure 5D). In contrast, let-7b mimics decreased TLR4 expression at protein level in a dose-dependent manner; whereas let-7b inhibitors upregulated the expression of TLR4 protein in AGS cells (Figure 5E, 5F). In GES-1 cells, TLR4 was also negatively regulated by let-7b mimics at the protein level (Figure 5G). These findings suggest that let-7b regulates the expression of TLR4 via post-transcriptional suppression.

### Regulation of TLR4 by let-7b Contributes to the Immune Responses of Gastric Epithelium against *H.pylori* Infection

**Figure 6:**
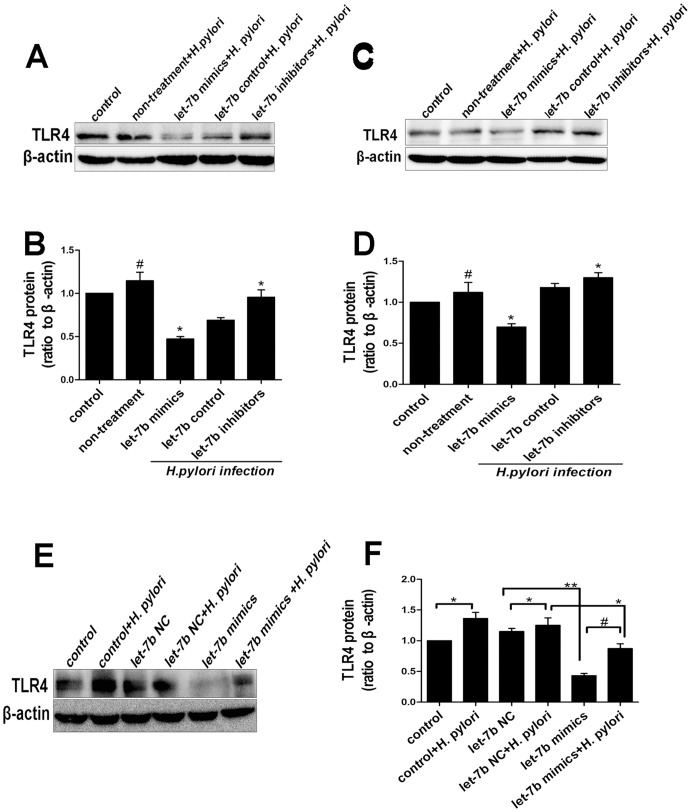
Let-7b regulates TLR4 contributing to the immune responses of gastric epithelium against *H.pylori* infection. (A–B) Effects of manipulating let-7b level on *H.pylori*-induced TLR4 expression in AGS cells as assessed by western blot and quantitative densitometric analysis. (C–D) Effects of manipulating let-7b level on *H.pylori*-induced TLR4 expression in GES-1 cells as assessed by western blot and quantitative densitometric analysis. AGS and GES-1 Cells were first transfected with let-7b control, let-7b mimics or let-7b inhibitors for 24 h, respectively, and then exposed to *H.pylori* 26695. The levels of TLR4 protein significant increased in cells with *H.pylori* infection compared with non-infection. Cells treated with let-7b inhibitors showed a further increase of TLR4 protein level following *H.pylori* infection. In contrast, transfection of cells with let-7b mimics diminished the increased expression of TLR4 protein induced by *H.pylori* infection. (E–F) *H.pylori* infection induced TLR4 expression, and overexpression of let-7b by mimics significantly decreased TLR4 protein level, irrespective of *H.pylori* infection. Data were expressed as mean ± S.D. from three independent experiments. *p<0.05, **p<0.01, #p<0.05.

To test the potential role of let-7b in regulating TLR4 expression in *H.pylori* infection, we assessed the influence of experimentally manipulating let-7b level on *H.pylori*-induced TLR4 upregulation in AGS and GES-1 cells. Cells were first transfected with let-7b control, let-7b mimics or let-7b inhibitors, respectively, and then exposed to *H.pylori* 26695. As shown in Figure 6A, 6B, 6C, 6D, we found a significant increase of TLR4 protein in cells with *H.pylori* infection compared with non-infection. Cells transfected with let-7b inhibitors showed a further increase of TLR4 protein level following *H.pylori* infection. In contrast, overexpression of let-7b by mimics diminished the increased expression of TLR4 protein induced by *H.pylori* infection. Therefore, *H.pylori* infection may upregulate TLR4 expression by downregulating let-7b expression in gastric epithelial cells. Moreover, this regulation effect of let-7b is accomplished via post-transcriptional suppression of TLR4 mRNA. In order to clarify the relationship between *H.pylori*, TLR4 and let-7b, we measured TLR4 protein by manipulating the overexpression of let-7b in AGS cells with *H.pylori* infection. As shown in Figure 6E and 6F, overexpression of let-7b by mimics significantly decreased TLR4 protein level irrespective of *H.pylori* infection.

### Overexpression of let-7b Decreases NF-κB Activity and Downregulates IL-8, COX-2 and CyclinD1

**Figure 7:**
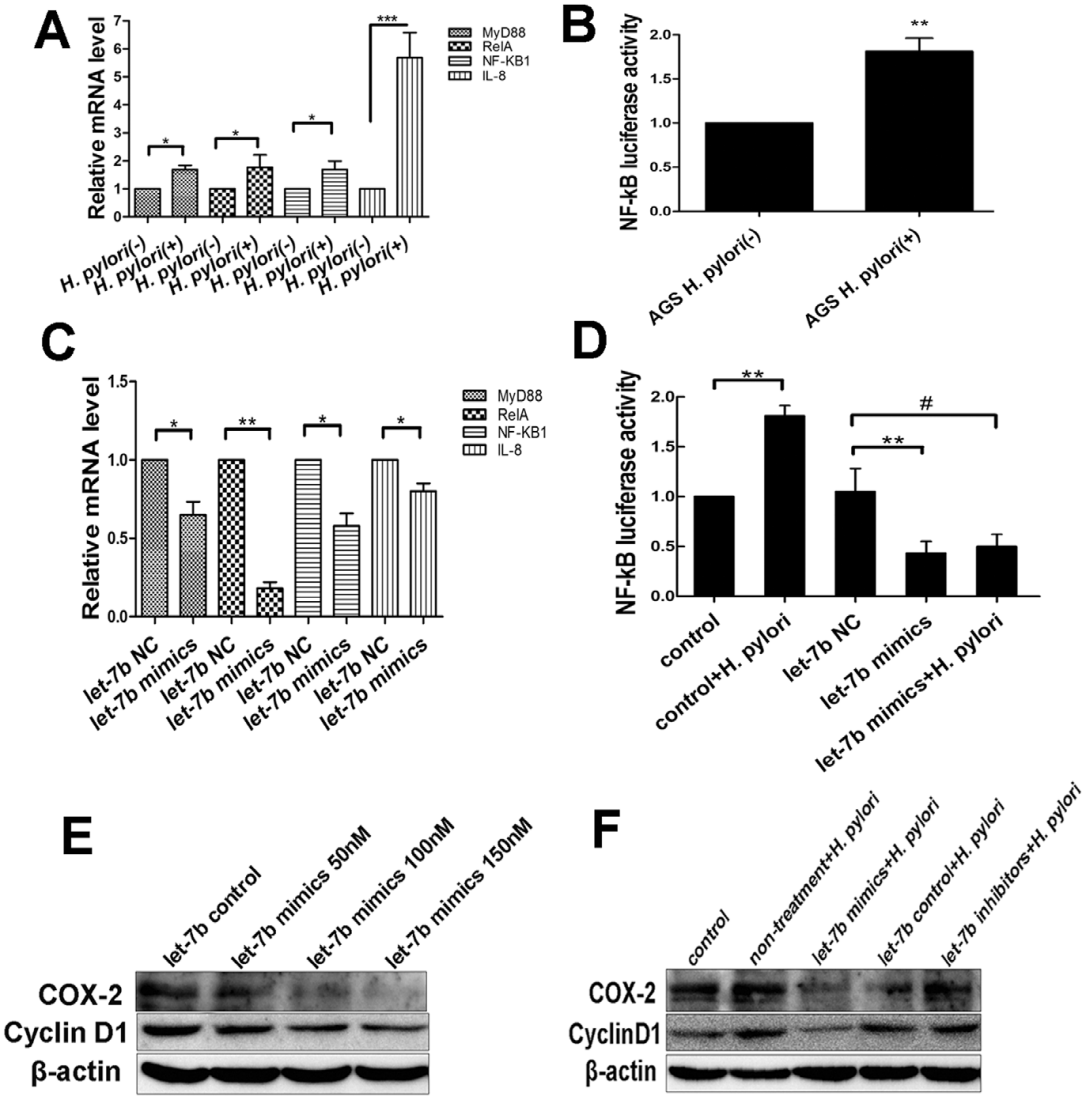
Overexpression of let-7b decreases NF-κB activity and downregulates COX-2, CyclinD1 protein expression. (A–B) *H.pylori* infection increased the expression of MyD88, p65/RelA, p50/NF-κB1 and IL-8 mRNA levels, and enhanced the NF-κB luciferase activity. (C–D) Overexpression of let-7b by mimics downregulated MyD88, p65/RelA, P50/NF-κB1 and IL-8 mRNA levels, and decreased NF-κB luciferase activity compared to let-7b negative control. Transfection with let-7b mimics inhibited NF-κB luciferase activity, irrespective of *H.pylori* infection. (E) Overexpression of let-7b by mimics decreased the expression of COX-2 and CyclinD1 in a dose-dependent manner. (F) Effects of let-7b on *H.pylori*-induced COX-2 and CyclinD1 expression in AGS cells were assessed by western blot. Data were expressed as mean ± S.D. from three independent experiments. *p<0.05, **p<0.01, ***p<0.001, # p<0.05.

Previous study has identified gastric epithelial cells as a source of IL-8 production [26]. *H.pylori* infection induced activation of NF-κB with the overexpression of mRNA levels of MyD88, p65/RelA, p50/NF-κB1 and IL-8 (Figure 7A, 7B). Overexpression of let-7b by mimics downregulated the mRNA levels of MyD88, p65/RelA and p50/NF-κB1, and decreased NF-κB activity as compared to let-7b negative control in AGS cells (Figure 7C, 7D). Therefore, let-7b may be involved in the inflammation and immune responses associated with *H.pylori* infection. However, overexpression of let-7b by mimics could inhibit *H.pylori*-induced the increase of NF-κB luciferase activity (Figure 7D).

Furthermore, overexpression of let-7b by mimics decreased the expression of the downstream genes. The expression of IL-8 mRNA was inhibited (Figure 7C), and the levels of cyclooxygenase-2 (COX-2) and CyclinD1 decreased in a dose-dependent manner (Figure 7E).

To test the role of let-7b in regulating the expression of the downstream genes of TLR4 in *H.pylori* infection, COX-2 and CyclinD1 proteins were determined. A significant increase of COX-2 and CyclinD1 in *H.pylori* infected cells was found compared with controls. Transfection with let-7b inhibitors in AGS cells showed a further increase of COX-2 and CyclinD1 expression following *H.pylori* infection. In contrast, overexpression of let-7b by mimics diminished *H.pylori*-induced the increase of COX-2 and CyclinD1 proteins (Figure 7F).

### TLR4 and NF-κB Signaling Pathway is Required for the Reduction of let-7b Expression in Response to *H.pylori* Infection

**Figure 8:**
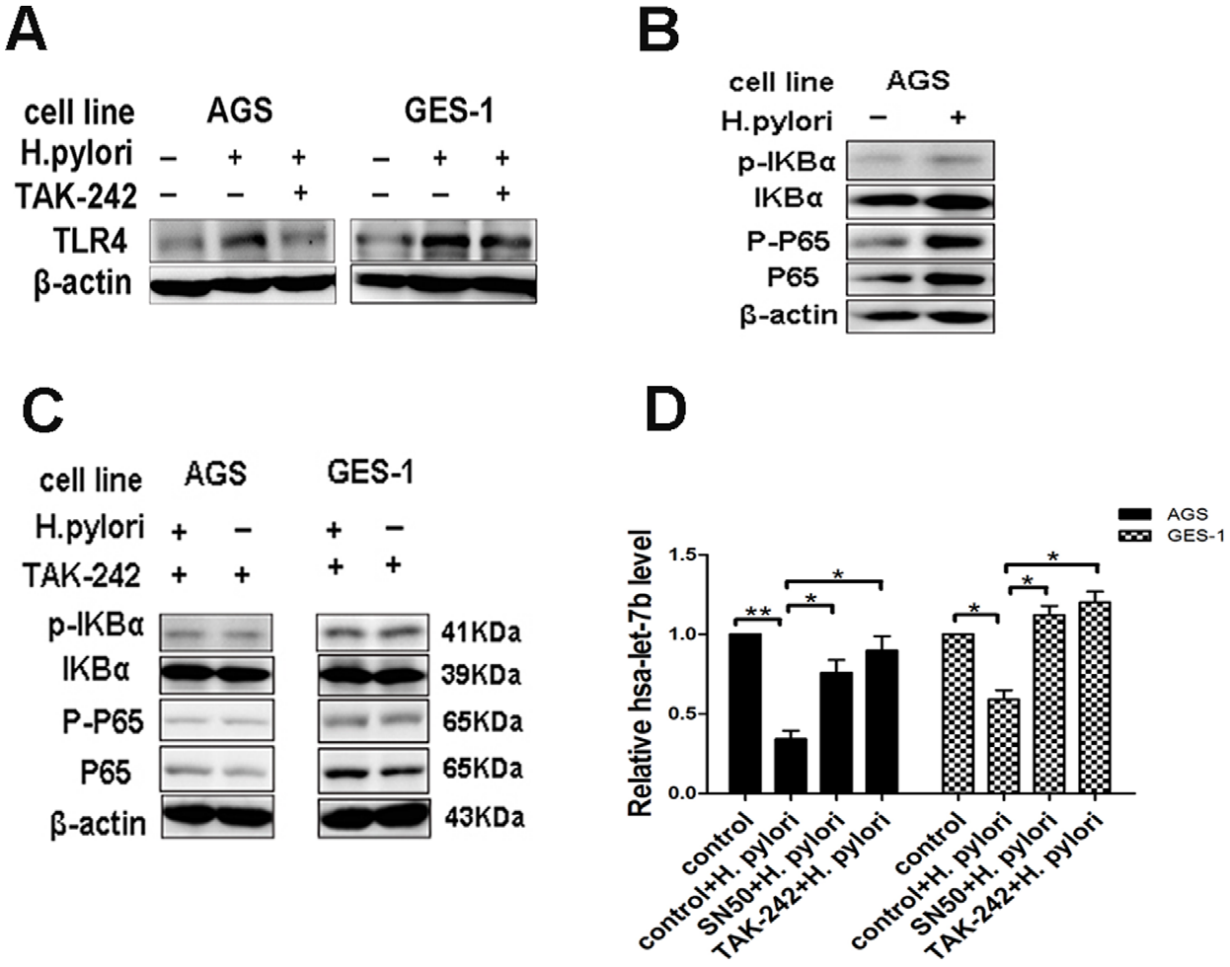
TLR4 and NF-κB signaling pathway is required for the reduction of let-7b expression in response to *H.pylori* infection. (A) TKA-242 (1 uM) decreased the expression of TLR4 protein in AGS and GES-1 cells with *H.pylori*-infection. (B) Infected with *H.pylori* 26695 for 1 h increased the phosphorylation of IKBα and NF-κB p65 in AGS cells. (C) Pretreatment of AGS and GES-1 cells with TKA-242 (1 uM) for 1 h significantly prevented the increase of IKBα and NF-κB p65 phosphorylation in response to *H.pylori* infection. (D) AGS and GES-1 cells were pretreated with or without TAK-242 (1 uM) or SN50 (50 ug/ml) for 1 h prior to *H.pylori* infection. The expression of let-7b was determined by qRT-PCR. All data were normalized by U6 snRNA. Data were expressed as mean ± S.D. from three independent experiments. *p<0.05, **p<0.01.

In order to determine whether TLR4 and NF-κB signaling pathway is required for the regulation of let-7b expression in response to *H.pylori* infection, we measured the expression of let-7b in AGS and GES-1 treated with either a specific TLR4 inhibitor (TAK-242, 1 uM) or a specific NF-κB inhibitor (SN50, 50 ug/ml). TAK-242 inhibited the expression of TLR4 in AGS and GES-1 cells in response to *H.pylori* infection (Figure 8A). As shown in Figure 8B, *H*.*pylori* infection increased the phosphorylation of IKBα and NF-κB p65 in AGS cells. Pretreatment of AGS and GES-1 cells with TKA-242 (1 uM) prevented the increase of IKBα and NF-κB p65 phosphorylation in response to *H.pylori* infection (Figure 8C). Furthermore, the levels of let-7b expression in cells pretreated with TAK-242 or SN50 were measured. Both TAK-242 and SN50 significantly inhibited *H.pylori-*induced downregulation of let-7b (Figure 8D). These data suggest that TLR4 and NF-κB signaling pathway is required for the reduction of let-7b expression in response to *H.pylori* infection.

## Discussion

*H.pylori* plays an etiologic role in the development of chronic gastritis, peptic ulceration and gastric adenocarcinoma [27]. In recent years, the role of miRNAs in pathogenesis of gastric cancers has been extensively studied. However, little is known regarding on the association between miRNAs and the immune and inflammatory responses in *H.pylori* infection. Our study indicates that TLR4 expression increases whereas let-7b expression decreases in *H.pylori* infection; let-7b mediates TLR4 expression via post-transcriptional regulation and inhibits NF-κB activity through MyD88 dependent pathway. Down-expression of let-7b is associated with the upregulation of TLR4, the activation of NF-κB, and the increased expression of COX-2 and CyclinD1 in *H.pylori* infection. These data suggest that let-7b may be involved in the upregulation of TLR4 in gastric epithelial cells in *H.pylori* infection, and contribute to the consequent initiation of innate immune responses and inflammation of gastric mucosa against *H.pylori* infection.

Gastric epithelial cells provide the first point of contact between *H.pylori* and the host. Studies have indicated that TLR4 expression is induced in gastric epithelial cells and initiates the innate immune responses to *H.pylori* [1], [2]. Recently, miRNAs have been proposed to participate in regulating the innate immune responses. Taganov et al. found that three miRNAs (miR-146a/b, miR-155 and miR-132) overexpressed in monocytic cells in response to TLR4 ligand lipopolysaccharide (LPS) treatment [28]. Moreover, miRNA-146 was in control of TLRs and cytokine signals through a negative feedback regulation loop involving downregulation of IRAK1 and TRAF6 [20], [28]. A recent study demonstrated that let-7i, a member of let-7 family, directly regulated TLR4 expression and contributed to the immune responses of cholangiocyte against *C.parvum* infection [17]. Another study suggested that atorvastatin downregulated TLR4 signal via let-7i expression in patients with coronary heart disease, possibly contributing to the benefit effects of atorvastatin on this disorder [29]. Other miRNAs, such as miR-511, miR-21, miRNA-223, miRNA-105 and let-7e were also reported to be involved in the regulation of TLRs signal [13], [30], [31]. Here, we found that let-7b was remarkably decreased in gastric epithelial cells after *H.pylori* infection. Let-7b mimics containing the 3′-UTR of TLR4 mRNA with the putative binding sites remarkably decreased the luciferase activity, indicating that TLR4 was a target of let-7b. Let-7b regulated TLR4 expression via post-transcriptional suppression in gastric epithelial cells. Treatment with let-7b mimics or let-7b inhibitors caused reciprocal modification of TLR4 protein expression with no significant alteration of TLR4 mRNA expression. However, a recent study reported that both mRNA and protein of TLR4 were decreased in THP-1 cells transfected with let-7i mimics [29]. The inconsistent results suggest that in different cells, let-7 family may regulate TLRs in different manners despite their high degree of homology.

NF-κB plays an important role in regulating the expression of genes involved in inflammation, cell proliferation and apoptosis [32], [33]. The activation of NF-κB involves the phosphorylation of IKKα, IKKβ and NIK, and the expression of TRAF2 and TRAF6 in intracellular signaling pathway after *H.pylori* infection [34]. Infection of *H.pylori* induces NF-κB activity in gastric epithelial cells, resulting in the expression of IL-8 [35]–[37]. When TLR4 is bound to bacterial LPS, it may recruit MyD88 and IRAK, and subsequently activates NF-κB [38], [39]. Infection of *H.pylori* induces TLR4 expression in gastric epithelial cells, thus activates NF-κB and results in the production of various cytokines [1], [4]. In our study, we found that infection of *H.pylori* activated NF-κB with the overexpression of MyD88, p65/RelA and p50/NF-κB1 in AGS cells. Overexpression of let-7b by mimics downregulated the expression of MyD88, p65/RelA and P50/NF-κB1, and decreased NF-κB activity. On the other hand, recent studies have revealed the possible role of NF-κB in regulating the expression of let-7. Wang et al. reported that NF-κB could regulate let-7a-3 promoter [40]. Activation of NF-κB induces the expression of Lin-28B [40], [41], a protein that blocks let-7 maturation, resulting in the inhibition of let-7 expression [40]–[43]. In our study, a specific TLR4 inhibitor (TAK-242) and a specific NF-κB inhibitor (SN50) significantly inhibited the *H.pylori-*induced downregulation of let-7b.

Gastric dysplasia presents a high level of TLRs expression, indicating that these receptors may play a role in the development of gastric adenocarcinoma [44]. Our study indicates that the expression of let-7b decreases in patients with *H.pylori* infection, and the expression of TLR4, MyD88, NF-κB, COX-2, CyclinD1 and IL-8 is upregulated. These results provide the evidence that some specific miRNAs may be involved in the persistent inflammation and the development of gastric adenocarcinoma induced by chronic infection of *H.pylori*. Since let-7 miRNAs generally plays a tumor-suppressive role, decreased expression of let-7b may promote the inflammatory responses and tumorigenesis associated with *H.pylori* infection. In this study, we found that let-7b overexpression could inhibit *H.pylori*-induced the increase of NF-κB activity. Therefore, overexpression of let-7b by gene transfection may inhibit the immune responses and inflammation induced by NF-κB, which is in favor of the inflammation control and cancer prevention after *H.pylori* infection.

In conclusion, the expression of let-7b decreases in *H.pylori* infected gastric mucosal samples and the cell lines. Upregulation of let-7b by mimics attenuates NF-κB activity and may be involved in regulating the native immune of gastric epithelium after *H.pylori* infection by targeting TLR4. TLR4 and NF-κB signaling pathway is required for the reduction of let-7b expression in response to *H.pylori* infection. These findings reveal the complicated signal transduction in the immune responses between bacteria and the host in *H.pylori* infection, and provide new ideas for the use of miRNAs in prevention and treatment of *H.pylori* related diseases.

## Materials and Methods

### Ethics Statement

The study was approved by the ethical committee of Peking University First Hospital. Written informed consent was obtained from each patient. The investigation conforms to the principles outlined in the Declaration of Helsinki.

### Patients and Gastric Mucosal Samples

Patients undergoing upper gastrointestinal endoscopy in Peking University First Hospital were enrolled. In total, 15 patients with *H.pylori*-induced chronic gastritis and 8 *H.pylori*-negative healthy control subjects were included. Infection of *H.pylori* were determined by rapid urease test, histology and ^13^C-urea breath test. Patients were regarded as being *H.pylori* positive if two of the tests were positive. Patients with atrophic gastritis, intestinal metaplasia and atypical hyperplasia were excluded. Three biopsy specimens were obtained from gastric antrum from each patient. One specimen was used for rapid urease test; the second was for histologic analysis; the third was frozen in liquid nitrogen immediately for RNA extraction. Histological assessment was performed by two pathologists who were blinded to the results of the experiments. Patients with liver disease, renal impairment, systemic infection, use of anti-secretory or nonsteroidal anti-inflammatory drugs (NSAIDs), tumors, alcohol abuse, drug addiction, pregnancy, and use of medications for *H.pylori* eradication during the preceding 4 weeks were also excluded from the study.

### Cell Culture and *H.pylori* Strains

Human gastric cancer cell line AGS and human embryonic kidney cell line HEK-293T were obtained from the American Type Culture Collection (Manassas, Virginia). An immortalized gastric epithelial cell line GES-1 [45] was a gift from Beijing Institute for Cancer Research (Peking University, Beijing, China). The AGS and the GES-1 cell lines were cultured in RPMI 1640 medium (Hyclone) supplemented with 10% fetal bovine serum (FBS, Gibco, USA) at 37°C in presence of 5% CO2. The HEK-293T cells was cultured in DMEM with 10% FBS (Gibco, USA) in the same condition. The wild type *H.pylori* 26695 (obtained from American Type Culture Collection) and its cagA mutant strain (ΔcagA 26695, a generous gift from professor Jianzhong Zhang, Chinese Center For Disease Control And Prevention) were grown in a microaerobic humidified atmosphere on Columbia agar (Oxoid, Cambridge, UK) plate with 8% defibrinogen goat blood at 37°C. After 48 h of culture, *H.pylori* was harvested in brain heart infusion and suspended at a concentration of 3×10^8^ colony-forming units (cfu)/ml in RPMI 1640. *H.pylori* bacteria were then cocultured with AGS, GES-1 at a ratio of 100 bacteria per cell [1], [18]. Cells were collected at 6 h, 12 h, 24 h and 48 h after *H.pylori* infection.

### RNA Extraction and Quantitative RT-PCR

Total RNA including miRNAs and mRNAs were isolated and purified by TRIzol reagent (Invitrogen, USA). Concentrations were measured using a NanoDrop spectrophotometer (ND-1000 V3.5.2 software, USA).

Reverse transcription of miRNAs to cDNAs were performed using Taqman MicroRNA Assays (Applied Biosystems, USA). 15 µL RT reaction volume consisted of 7 µL master mix, 3 µL of 5 µM RT primer (Ribobio, China), and 5 µL RNA sample (50 ng total RNA). Reverse transcription was performed at 16°C for 30 minutes, 42°C for 30 minutes, 85°C for 5 minutes on thermal cycler (Eppendrof, Germany).

Reverse transcription of mRNAs to cDNAs were performed using High Capacity cDNA Reverse Transcription Kits (Applied Biosystems, USA). 20 µL RT reaction volume consisted of 10 µL 2×RT master mix and 10 µL RNA sample (1 µg total RNA). Reverse transcription was performed in a thermal cycler (Eppendrof, Germany) at 25°C for 10 min, 37°C for 120 min, 85°C for 5 min. The synthetic cDNAs were stored at −70°C.

Real-time PCR was performed using the Applied Biosystems 7300 Sequence Detection System. Primers were designed by Primer Express 3.0 software (Applied Biosystem, Foster City, USA) and were synthesized from Tianyi Huiyuan Bioscience (Beijing, China) (Table 1). 15 µL real-time PCR reaction mixture included 1 µL of cDNA, 7.5 µL of Power SYBR Green PCR Master Mix (Applied Biosystems, USA), 2 µL of forward and reverse primers and 4.5 µL of water. The reactions were carried at the following parameters: predenaturation at 95°C for 10 min, followed by 40 cycles at 95°C for 15 sec, 60°C for 1 min, and a further melting curve step at 95°C for 15 sec, 60°C for 30 sec, 95°C for 15 sec. U6 snRNA or GAPDH expression was assayed for normalization. All reactions were performed in triplicate. The relative expression levels were determined with the ΔC_T_ method and reported as 2^−ΔΔCT^.

**Table 1:** Sequence of primers used for real-time PCR.

Gene name	Primer sequence	Size
TLR4-F	5′-CTTGGACCTTTCCAGCAACAA	105 bp
TLR4-R	5′-GGGTTCAGGGACAGGTCTAAAA	
MyD88-F	5′-TGACTTCCAGACCAAATTTGCA	95 bp
MyD88-R	5′-GGAACTCTTTCTTCATTGCCTTGT	
RelA-F	5′-TTCCCGCAGCTCAGCTTCT	75 bp
RelA-R	5′-TTGATGGTGCTCAGGGATGA	
NF-kB1-F	5′-CCTCCACAAGGCAGCAAATAG	85 bp
NF-kB1-R	5′-CTGAGTTTGCGGAAGGATGTCT	
IL8-F	5′-ACACTGCGCCAACACAGAAAT	85 bp
IL8-R	5′-CCTCTGCACCCAGTTTTCCTT	
GAPDH-F	5′-GCCTGGTCACCAGGGCT	121 bp
GAPDH-R	5′-AATTTGCCATGGGTGGAATC	

### Immunohistochemistry

Tissue specimens for immunohistochemical analyses were fixed in 10% formalin and paraffin-embedded. Sections were subjected to routine deparaffinization and rehydration. The endogenous peroxidase activity was inhibited by incubation with 3% hydrogen peroxide for 10 min. Antigen retrieval was achieved by microwaving in 0.01 mol/L citrate buffer (pH 6.0) for 25 min, and then cooling to room temperature. After three PBS washes, the specimens were incubated with mouse anti-human TLR4 (1∶75, Abcam, USA) at 4°C overnight. After incubation with anti-mouse IgG horseradish peroxidase (1∶100, Dako, Denmark) for 1 h, signals were developed with 3,3^,^-diaminobenzidine (DAB). The sections were then counterstained with hematoxylin and mounted.

Ten fields of vision per section (three to four sections per gastric specimen) were observed blindly at 20× as a semiquantitative assessment for immunohistochemical staining. The TLR4 staining were evaluated with the Image Pro Plus analysis software (version 6.0; Dallas, TX, USA). The positive signals were quantified as the mean optical density (integrated option density/area).

### Western Blot

Cells were lysed with RIPA buffer (Sigma, USA) containing a mixture of protease inhibitor cocktail kit (thermo, USA) for 30 min. Then the lysates were centrifuged at 4°C, 12,000 rpm for 15 min. The concentrations of proteins were measured by BCA protein assay kit (pierce, USA). Then the proteins were boiled at 95°C for 5 min and stored at −70°C. 50 µg proteins were subjected to electrophoresis in 10% sodium dodecyl sulfate polyacrylamide gels and transferred onto PVDF membrane (Amersham Biosciences, USA). The PVDF membranes were blocked for 1 h with 5% non-fat milk (BD, USA) at room temperature. Membranes were probed with mouse monoclonal antibody against human TLR4 (Santa Cruz, USA) at a dilution of 1∶100 at 4°C overnight, and then washed three times with TBST. The secondary antibody was added and conjugated with horseradish peroxidase (Santa Cruz, USA) at a dilution of 1∶2000 for 1 h. The membrane was washed three times with TBST and enhanced chemiluminescence (pierce, USA) was used to detect the antigen on X-film (Kodak Image station 4000 mm pro, Japan). The blots were stripped using stripping buffer for 20 min at 60°C and reprobed using rabbit polyclonal antibody against human CyclinD1 (Santa Cruz, USA) at a dilution of 1∶300 and polyclonal antibody against COX-2 (Santa Cruz, USA) at a dilution of 1∶150. The secondary antibody was added (goat anti-rabbit antibody for CyclinD1, donkey anti-goat antibody for COX-2, Santa Cruz, USA) and conjugated to horseradish peroxidase. β-actin monoclonal antibody (Immunocreat, USA) was used to monitor protein loading at a dilution of 1∶1000. P-65, p-P65, IKBα, p-IKBα (obtained from CST, USA) antibodies were used at a dilution of 1∶1000. Expression of TLR4, COX-2 and CyclinD1 were quantified relative to β-actin expression with Image software (National Institutes of Health, USA).

### Cell Transfection

AGS and GES-1 cells were seeded in 6-well plates and transfected at the percentage of sixty confluences (approximately 3×10^5^ cells). The oligonucleotides of hsa-let-7b mimics (5′-UGAGGUAGUAGGUUGUGUGGUU-3′, antisense 5′-CCACACAACCUACUACCUCAUU-3′), has-let-7b inhibitors (5′-AACCACACAACCUACUACCUCA-3′), and scrambled has-let-7b negative control (5′-UUCUCCGAACGUGUCACGUTT-3′, antisense 5′-ACGUGACACGUUCGGAGAATT-3′) were synthesized from GenePharma (shanghai, China). Transfections were performed using Lipofectamine 2000 transfection reagent (Invitrogen). The two kinds of cells were respectively transfected with 100 nM let-7b mimics, let-7b inhibitors, and scrambled let-7b negative control. Total RNA was extracted at 24 h after transfection. Total protein was isolated at 48 h after transfection.

### Luciferase Reporter Constructions and Luciferase Assay

The wild-type 3′-UTR of human TLR4 holding let-7b binding sites (TLR4-3′-UTR-F: 5′-AGCTTTGTTTAAACAGAGGAAAAATAAAAACCTCCT -3′, TLR4-3′-UTR-R: 5′-GAATGCGGCCGCTTGAGAGAGAGAAAGAAAGAG-3′) and the mutant containing the seed region (TACCTCA to ATGGAGT) (TLR4-3′-UTR-Mut-F: 5′-AACTCTATGGAGTTCAAGTTGAATAAAGACAG -3′, TLR4-3′-UTR-Mut-R: 5′- CTTGAACTCCATAGAGTTGAAATGACTTTCTT -3′) were PCR amplified from genomic DNA of HEK-293T cells. The amplified PCR products were then extracted and purified with a Genomic DNA Extraction kit (Tiangen, China). The PCR products were digested and inserted into the pmiR-RB-REPORT™ vector (Ribobio, China) between the XhoI and NotI sites using restriction enzyme and Phusion DNA polymerase (Fermentas, USA). Finally, the constructed luciferase reporter plasmids were transferred into Escherichia coli (DH5α, tiangen, China). The wild-type and the mutant sequences were validated by DNA sequencing.

Then, HEK-293T cells were seeded in 24-well plates (approximately 6.25×10^4^ cells/well) and transfected with 400 ng of plasmids (wild-type or mutant) and 100 nM miRNA (let-7b mimics or let-7b negative control [NC]).

The pNF-κB-luc (PathDetect® cis-Reporting Systems, Stratagene, USA) containing NF-κB (5×) enhancer element sequence (TGGGGACTTTCCGC) was used to determine the NF-κB luciferase activity. AGS cells were cotransfected with 0.4 µg of firefly reporter luciferase vector (pNF-KB-luc), 0.02 µg of Renilla luciferase control vector (Promega) with lipofectamine 2000 (Invitrogen). 48 h after transfection, luciferase activity was measured using Dual-luciferase reporter assay kit (Promega, USA) according to the protocol. Luminescence intensity was read with a Microplate Luminometer (Vertias™, Turner Biosystems, USA). Transfection experiments were independently repeated three times.

### Statistical Analysis

The fold changes of gene expression was calculated by the equation 2^−ΔΔCT^ [46]. Statistical analysis was performed using SPSS 16.0. Values were expressed as means ± S.D. Differences between groups were calculated with two-talied Student’s t-test or nonparametric Wilcoxon test and Mann–Whitney U test. All analyses were considered as significant when *p*<0.05.
